# Exploring the personality and relationship factors that mediate the connection between differentiation of self and phubbing

**DOI:** 10.1038/s41598-024-55560-1

**Published:** 2024-03-19

**Authors:** Ora Peleg, Meyran Boniel-Nissim

**Affiliations:** Departments of Educational Counseling and Education, The Max Stern Yezreel Valley College, Yezreel Valley, Israel

**Keywords:** Differentiation of self, Fear of missing out, Romantic relationship satisfaction, Loneliness in intimate relationships, Phubbing, Psychology, Risk factors

## Abstract

One of the family patterns crucial for mental and physical well-being is differentiation of self. In this study, our aim was to investigate its impact on the phenomenon of phubbing—where individuals prioritize smartphones over in-person interactions. The prevalence of phubbing behavior has risen substantially in tandem with the increased adoption of smartphones. The study investigated familial, interpersonal, and personal factors that could potentially contribute to the escalation of phubbing behaviors. It was hypothesized that differentiation of self would be associated with phubbing through the mediation of fear of missing out, romantic relationship satisfaction, and loneliness in intimate relationships. We also expected gender differences in the study variables. A sample of 431 young adults, with an average age of 29 (M = 29.05, SD = 9.14), completed the DSI-R, Phubbing, FoMO, ENRICH, and LIRS questionnaires online. Results indicated that fear of missing out mediated the relationship between three dimensions of differentiation of self—emotional reactivity, emotional cutoff, and fusion with others—and phubbing behavior. However, the expected mediation by romantic relationship satisfaction and loneliness in intimate relationships did not reach significance, although these variables were found to be associated with differentiation of self. Women reported higher levels of phubbing behavior, emotional reactivity, and fusion with others, whereas men reported higher levels of I-position. We conclude that fear of missing out may serve as a catalyst, triggering anxiety in individuals, which in turn drives them to adopt phubbing as a coping mechanism. Moreover, individuals with lower levels of differentiation of self appear to be at increased risk of engaging in phubbing behaviors through the mediation of fear of missing out.

## Introduction

Smartphones have become ubiquitous, seamlessly integrating into every aspect of modern life^1^. This pervasive integration has led individuals to prioritize smartphone engagement over face-to-face interactions, a trend that has sparked significant research interest^2,3^. As a result, there is a critical need to understand the profound implications of this digital intrusion on personal connections and human relationships, particularly within romantic contexts^4,5^.

The universal use of smartphones has given rise to a new interpersonal phenomenon known as “phubbing”, a portmanteau of “phone” and “snubbing”. Phubbing describes the act of diverting attention to a smartphone during face-to-face interaction, often at the expense of immediate social engagement^6^. This behavior has emerged as a significant source of conflict and dissatisfaction within romantic relationships^7,8^. Studies have linked elevated phubbing to fear of missing out (FoMO), decreased relationship satisfaction, and increased feelings of loneliness^9–11^.

One crucial factor influencing relationship dynamics and personality characteristics is differentiation of self (DoS), which involves maintaining personal identity while fostering stable relationships^12^. Research has shown the significant impact of DoS on mental well-being and romantic relationship satisfaction, as well as its association with self-confidence and emotional regulation^13,14^. Phubbing, characterized by smartphone-induced distractions during face-to-face interactions, is linked to lower self-esteem, decreased relationship satisfaction, increased loneliness, and disrupted family dynamics^1–4^, as well as psychological problems^15^. Phubbing also strains intergenerational relationships, exacerbating tension when younger family members engage in this behavior during encounters with older adults^5^. Yet, the association between DoS and phubbing remains underexplored. Understanding how DoS is related to phubbing can shed light on digital communication behaviors and the familial and personality factors that may increase it. Hence, the current study explores the contribution of DoS to phubbing, as mediated by fear of missing out (FoMO), romantic relationship satisfaction, and loneliness in intimate relationships.

### Exogenous variable: differentiation of self

In a broad sense, DoS entails an individual’s aptitude to define their identity without being flooded by emotions and to foster profound, stable relationships. Its theoretical construct encompasses four dimensions. I-position evaluates the capacity to uphold one’s personal needs, desires, and thoughts, even when these diverge from the viewpoints of close associates. Emotional reactivity signifies the inclination to respond to stress-inducing triggers automatically and emotionally. Emotional cutoff characterizes the tendency to isolate oneself physically, emotionally, and verbally, distancing from sharing emotions and resorting to rigid detachment from loved ones as a coping mechanism in times of stress within intimate relationships. Finally, fusion with others entails forming symbiotic connections and seeking validation from significant others. Interpersonally, DoS denotes the individual’s adeptness at harmonizing intimacy and autonomy; intrapersonally, it signifies the capacity to cope with stress calmly^15^.

Gender differences have been found in DoS dimensions. Higher levels of emotional reactivity and fusion with others were observed among women and elevated levels of emotional cutoff among men. It has been suggested that women often engage in closer relationships and express more emotions, while men tend to repress their feelings, particularly when under stress (e.g.^12^).

DoS has been identified as a pivotal personality pattern contributing to mental well-being (e.g.^12^). Well-differentiated individuals demonstrate proficient relationship management alongside increased levels of functioning, adaptability, and self-efficacy^15,16^. Poorly differentiated individuals exhibit elevated emotional distress and anxiety^17^ and diminished romantic relationship satisfaction^18^.

Numerous studies have delved into the interplay between DoS and romantic relationship satisfaction (e.g.^19^). Peleg’s^20^ study found an inverse correlation: lower emotional cutoff was linked to heightened romantic relationship satisfaction. Notably, gender differences also emerged. Men’s satisfaction was associated with I-position, emotional reactivity, and emotional cutoff, while women’s satisfaction correlated mainly with emotional cutoff. In a recent study, DoS positively predicted romantic relationship satisfaction, influencing it indirectly through touch, affirmation, quality time, and gifts^21^. In addition, DoS predicted improved relationship quality, stability, and reduced attachment issues in individuals in the U.S. Over time, DoS led to improved relationship quality and decreased anxious attachment for Spanish individuals, while benefiting U.S. couples with greater stability and reduced anxious and avoidant attachment^16^. Higher DoS in wives has also been related to lower fear of intimacy, impacting higher romantic relationship satisfaction in husbands^22^. Furthermore, romantic relationship satisfaction partially mediated the association between DoS and forgiveness and exit/neglect, and DoS directly impacted romantic relationship dynamics^23^. These findings align with Bowen’s theory, underscoring the role of DoS in intimacy and its influence on romantic relationship satisfaction^14^. While existing research has explored the connection between partners’ DoS and romantic relationship satisfaction, this has not been investigated in the context of phubbing.

Notably, the impact of DoS extends to various emotions and significant relationships. One study found that individuals who have difficulties establishing clear boundaries between “self” and “other” and engage in emotionally detached relationships with parents are more susceptible to parent-related loneliness^24^. Another study noted significant associations between DoS, loneliness, and divorce inclination. Attachment styles, mediated by DoS, predicted both loneliness and the likelihood of divorce. These results highlight the substantial mediating role of DoS in the connection between attachment styles, loneliness, and the tendency to divorce^25^. However, the direct relation between DoS and loneliness in intimate relationships remains unexplored.

While there is no explicit mention of a connection between DoS and FoMO, studies have established associations between parenting styles, self-esteem, and FoMO in adolescents and young adults^26^, as well as connections between parenting styles, self-esteem, and DoS^27^. Similarly, while there is no evidence of a direct association between phubbing and DoS, phubbing has been found to be linked to the parent–child relationship, which is an integral component of DoS^28^.

The investigation into the link between differentiation of self (DoS) and phubbing is crucial, considering the adverse effects of phubbing on aspects such as self-esteem, relationship satisfaction, loneliness, and family dynamics^1–4^. Phubbing has been linked to disrupted family patterns and estrangement from close relationships^4^. Thus, understanding how low DoS contributes to smartphone-induced distractions (i.e., phubbing) sheds light on the mechanisms shaping digital communication behaviors and their impact on individual well-being^5^.

### Endogenous variable: phubbing

The concept of “partner phubbing” denotes how individuals allow smartphone distractions to permeate their in-person interactions with their partners. This encapsulates scenarios where one’s romantic companion frequently disrupts communication, prioritizing prolonged smartphone engagement^29^. Research has shown that phubbing behavior is common among adults, with prevalence rates ranging from 35.5 to 47.2%. The prevalence of phubbing varies depending on such factors as age, education level, and cultural context. Overall, research suggests that phubbing is a widespread behavior that can have negative consequences on relationships and mental health^30^. Such behavior will likely erode couple dynamics, leaving individuals feeling undervalued^31^. As for gender differences in phubbing behavior, a study of 243 male and female college students aged 17–26 found that males tended to engage in phubbing more than females^32^. These findings are supported by additional studies (e.g.^33^).

The association between phubbing and personality characteristics can be elucidated through the framework of Uses and Gratifications Theory. This theory posits that individuals fulfil certain needs by using social media. From this perspective, individuals resort to social media to address such needs as sustaining interpersonal relationships, seeking entertainment, and garnering admiration^34–36^.

Several studies have linked phubbing to various psychological dimensions. For instance, partner phubbing has been shown to affect relationship satisfaction both positively and negatively in romantic relationships^29^. While smartphones and social media aid long-distance partners in staying connected^37^, excessive smartphone use has been associated with reduced intimacy and emotional sharing in long-distance relationships^31,38^, and escalating smartphone presence in romantic contexts can lead to frustration, alienation, and decreased relationship satisfaction, potentially triggering depressive symptoms^1^. Experimental studies have demonstrated that increased phubbing diminishes the perceived quality of communication^33^ and negatively impacts romantic relationship satisfaction^31^.

Notwithstanding the recent focus on partner phubbing, there is still uncertainty about the drivers of phubbing behaviors and the potential susceptibility of specific individuals to its more severe forms^39^. Research has shown that the connection between romantic relationship satisfaction and phubbing gains significance when influenced or moderated by variables such as family patterns^29^. Moreover, it has been observed that individuals may resort to phubbing their partners in response to heightened anxiety stress and FoMO^7^, aiming to alleviate negative emotions^40,41^.

### Mediating variables: fear of missing out, romantic relationship satisfaction, and loneliness in intimate relationships

FoMO, romantic relationship satisfaction, and loneliness in intimate relationships are proposed as mediating variables in the relationship between DoS and phubbing. FoMO is characterized by a persistent apprehension that others are engaging in enjoyable experiences from which one is excluded. Essentially, it signifies the anxiety stemming from not participating in meaningful and pleasurable activities in which others are currently involved^42^. Research has underscored that FoMO encapsulates apprehension of being disconnected and left out from various events, predominantly within social spheres^43^. The pervasive use of social media, providing real-time access to a wealth of information, has stoked this anxiety, propelling heightened smartphone and social media engagement and consequently fostering an increase in phubbing behavior^44^. Results regarding the association between FoMO and gender are varied. Earlier studies indicated small effects on gender differences, with women scoring higher than men (e.g.^45,46^). However, a recent study investigating the relationships between FoMO, age, and gender found no gender differences in FoMO^47^.

The association between FoMO and phubbing has been a focal point in a few studies. For instance, Davey et al.^7^ identified FoMO as a significant predictor of phubbing behavior among young adults in India. They suggested that passive engagement with social media applications could contribute to this connection. Similarly, it was observed that FoMO precedes phubbing tendencies among adolescents^48,49^. These study findings indicate that FoMO might act as a catalyst, prompting individuals to experience anxiety and leading them to resort to phubbing as a coping mechanism. Yet, the act of phubbing can engender a sense of detachment from reality, transporting individuals into a virtual realm. This tendency can impact an individual’s psychological well-being, particularly if they struggle to navigate this phenomenon^50^. FoMO is posited to mediate the relationship between DoS and phubbing due to its role in driving smartphone use and social media engagement^1^. Given that this taps into anxiety, and DoS has been related to various anxieties^12^, FoMO is expected to amplify this association.

Phubbing may also be related to satisfaction with romantic relationships. A stable and healthy relationship is often perceived as the cornerstone of happy individuals and well-adjusted families. Romantic relationship satisfaction refers to individuals’ favorable or unfavorable experiences within their romantic relationships^51^. It also encompasses “the degree to which spouses perceive that their partners meet their needs and desires”^21^, p. 388. In order to cultivate a mutually satisfying relationship, physical presence is insufficient; a meaningful connection between partners is essential, and both must be fully engaged^52^.

In a review of gender differences in the development of relationship satisfaction, Bühler et al.^53^ emphasize the need to consider gender as a moderator. However, findings are mixed; a meta-analysis by Jackson et al.^54^ suggests slightly lower satisfaction for women, but longitudinal studies (e.g.^55^) show varied trajectories, with some studies^56^ reporting no significant gender disparities.

A suitable framework for examining the impact of phubbing on human relationships and romantic relationship satisfaction is Media Displacement Theory^57^, which posits that individuals have limited time and attention, and that engaging in one communication activity can curtail engagement in other interpersonal interactions. This effectively reduces the time available for communication as new communication technologies emerge. This theory can explain scenarios where individuals have restricted time and attention for their daily routines^58^, such that devoting time to devices like cell phones may diminish opportunities for meaningful interactions between partners and potentially lower the overall quality of the relationship.

The disruptions caused by phubbing have the potential to undermine essential human needs for both personal agency and emotional attachment, consequently leading to a decline in the quality of romantic relationships among couples. This occurs as individuals perceive their partners to be emotionally distant. Indeed, phubbing has been found to impact closeness, connection, and the quality of conversations, as well as to be detrimental to interpersonal relationships, particularly when conversations involve personal topics^59^. Romantic relationship satisfaction is proposed as a mediating variable between DoS and phubbing because research has indicated that individuals with higher levels of relationship satisfaction tend to engage less in phubbing behaviors, prioritizing meaningful interactions with their partners over smartphone use^5,6^, and typically exhibit higher levels of DoS^20^.

It has been reported that lonely individuals tend to perceive lower partner regard and responsiveness, leading to lower relationship satisfaction and commitment^60^. Loneliness is the degree to which an individual lives in solitude and lacks significant interpersonal connections with others^61^. This distressing and subjective emotion arises when individuals perceive their social interactions as unsatisfactory and insufficient. In contrast to the past stereotype associating loneliness with older people, recent research suggests the experience encompasses a broad spectrum of age groups, including young adults, who often seek support through their smartphones^62^. It was reported that when individuals experience loneliness, they often seek ways to alleviate this emotion, such as social media applications (e.g., Facebook and Instagram) and messaging platforms (e.g., WhatsApp). While these devices offer a means to alleviate isolation, their excessive usage can be detrimental to the quality of human interactions^63^.

Gender differences in loneliness in intimate relationships have been explored in several studies. Schobin^64^ found that women tend to experience more relational loneliness (loneliness within the broader peer group), while men tend to experience more intimate loneliness (within a dyadic relationship with a friend). Another study, conducted among married individuals between the ages of 20 and 49^65^, tested the assumption that women more willingly acknowledge their loneliness, but this assumption was refuted. Overall, gender differences in loneliness in intimate relationships vary depending on the specific context and age group under study.

Evidence indicates that loneliness is also associated with biased perceptions of partner regard, communal motivation, and support provision, which in turn contribute to lower relationship satisfaction, commitment, disclosure, support, and happiness^66^. Loneliness and rejection sensitivity were found to be positively associated with threat sensitivity in romantic relationships, and rejection sensitivity mediated the relationship between loneliness and threat sensitivity^67^. Overall, these findings suggest that loneliness can compromise the quality of romantic relationships and contribute to mental health problems^68^.

Loneliness has been associated with elevated levels of FoMO, especially among those extensively involved in social media activities^69^, leading individuals to check their smartphones frequently to avoid social exclusion^70^. The fear of loneliness within relationships drives smartphone use, potentially increasing FoMO and phubbing behaviors^5,71^. Loneliness in intimate relationships is suggested as another mediating variable due to its potential to drive excessive smartphone use as a coping mechanism for social isolation^7^, inadvertently increasing phubbing behaviors^8,9^. It stands to reason that lower levels of DoS may exacerbate feelings of loneliness in light of the link between loneliness, phubbing, and distant family and marital relationships, coupled with findings that highlight the significant impact of the family environment on adolescent loneliness—with family functioning, parenting style, and the parent–child relationship all negatively predicting it^72^. Such exacerbated feelings of loneliness could ultimately contribute to elevated levels of phubbing in intimate relationships.

### Young adulthood

The current study focused on young adults, a demographic of particular significance in developmental psychology. Recent research underscores a notable shift in the understanding of the transition from childhood to adulthood by developmental psychologists, with societal and economic changes leading to delays in achieving traditional adult milestones, such as marriage, parenthood, and homeownership^73,74^. Arnett^75^ highlights the diverse experiences encountered during this transition period, as young individuals explore different paths before establishing a clear life direction. Furthermore, individuals in this phase often face challenges in emotional, interpersonal, familial, and romantic realms, sometimes resulting in various emotional disorders.

Research on this age group is also important due to their extensive use of the Internet and high dependence on smartphones—a phenomenon that has received less attention in this context^13^. Exploring phubbing in this age group can play a pivotal role in increasing awareness about the issue and promoting responsible smartphone use.

### The current study

**Figure 1:**
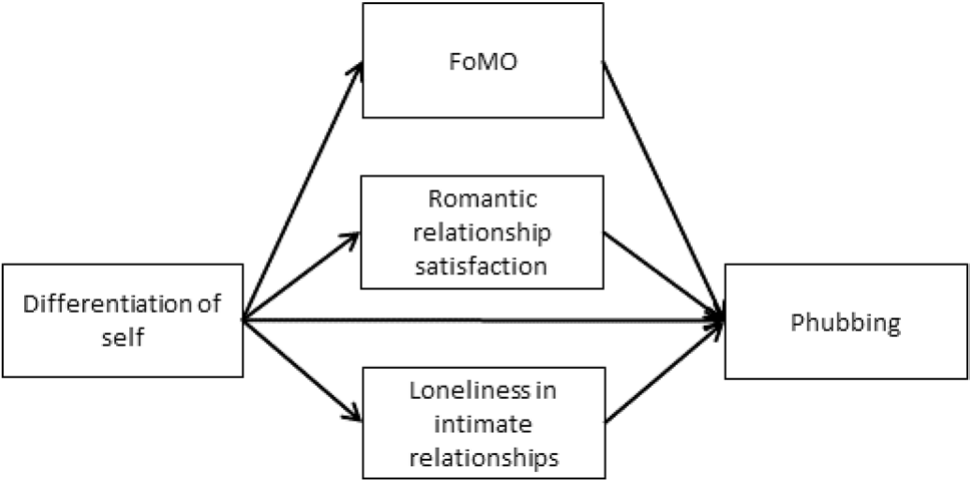
Research hypotheses model.

Based on the theories and empirical results discussed, sufficient evidence suggests that a partner’s use of a smartphone while in the company of their romantic partner may impact relationship satisfaction. With the increasing prevalence of cell phone usage among young adult couples^76^, it is crucial to examine the family, romantic relationship, and personal factors contributing to the rise of phubbing. FoMO, romantic relationship satisfaction, and loneliness in intimate relationships are proposed as mediating variables in the relationship between DoS and phubbing, with each variable playing a distinct role in amplifying the association between these constructs. This proposed mediation model seeks to provide a comprehensive understanding of the mechanisms underlying the relationship between DoS and phubbing behavior. Consequently, our primary objective was to explore whether a low level of DoS could augment FoMO, erode romantic relationship satisfaction, and amplify feelings of loneliness within intimate relationships, and subsequently increase phubbing behavior. Notably, each of the three mediating variables may contribute to phubbing. Exploring all three together may offer a more complete, in-depth picture that helps to discern and understand the relationship between DoS and phubbing. Hence, we hypothesized that DoS (I-position, emotional reactivity, emotional cutoff, and fusion with others) would be associated with phubbing through the mediation of FoMO, romantic relationship satisfaction, and loneliness in intimate relationships (Hypothesis 1, Fig. 1). Furthermore, drawing from previous findings, we anticipated that women would report higher levels of emotional reactivity, fusion with others, and FoMO, while men would report higher levels of emotional cutoff and phubbing (Hypothesis 2).

## Methods

### Participants

Study participants were 431 Israeli adults, about two-thirds of whom were females, with a mean age of 29 years (M = 29.05, SD = 9.14). We selected young adults for our sample because, at this age, they tend to be involved in romantic relationships, and many of them have established stable partnerships. Most were Jewish, heterosexual, secular, and employed. About a third were married, while the rest were cohabiting. Close to 25% had children. About 70% had a college education. The inclusion criteria were that all participants possessed smartphones and were involved in a romantic relationship. See Table 1 for more details.

**Table 1:** Sociodemographic and background characteristics (N = 431).

Variable	Categories	Values
Age, M (SD), range		29.05 (9.14), 18–60
Relationship duration, M (SD), range		6.06 (7.06), 1–47
Gender, n (%)	Male	150 (34.8)
	Female	281 (65.2)
Ethnicity, n (%)	Jewish	403 (93.5)
	Arab	28 (6.5)
Relationship status, n (%)	Married	134 (31.1)
	Cohabiting	297 (68.9)
Sexual orientation, n (%)	Heterosexual	396 (91.9)
	LGBTQ	35 (8.1)
Parenthood, n (%)	Yes	103 (23.9)
Number of children, n (%) (n = 103)	1	25 (24.3)
	2	42 (40.8)
	3	33 (32.0)
	4 +	3 (2.9)
Religiosity, n (%)	Secular	363 (84.2)
	Traditional	45 (10.4)
	Religious	23 (5.3)
Level of education, n (%)	High school diploma	125 (29.0)
	Undergraduate student	168 (39.0)
	College degree^a^	138 (32.0)
Employment, n (%)	Yes	323 (74.9)

^a^Includes graduate students.

### Instruments

The *Differentiation of Self-Revised *scale (DSI-R^77,78^) was translated into Hebrew and adapted to the Israeli context^20,79^. The questionnaire comprises 46 items distributed across four subscales: I-position, emotional reactivity, emotional cutoff, and fusion with others (sample item for emotional reactivity: “People have remarked that I’m overly emotional”). Respondents rate each item on a Likert scale ranging from 1 (not at all like me) to 6 (very much like me). Subscale scores are derived by averaging mean scores. Higher DoS is indicated by higher means for I-position and lower means for emotional reactivity, emotional cutoff, and fusion with others. Cronbach’s alpha coefficient demonstrated strong reliability for I-position (0.81), emotional reactivity (0.88), and emotional cutoff (0.81), and acceptable reliability for the fusion with others subscale (0.79).

The *Phubbing Questionnaire*^80^ was translated into Hebrew for the current study, using back translation. The questionnaire comprises ten items and two scales. The first scale pertains to communication disorders and is composed of five items (sample item: “People complain that I mess with my cell phone”). The second scale concerns obsession with the mobile phone (sample item: “I feel anxious if my phone is not nearby”). Answers are rated on a Likert scale ranging from 1 (never) to 5 (always). Cronbach’s alpha for the entire sample in the current study was 0.78.

The *Fear of Missing Out (FoMO)* scale^81^ was translated into Hebrew and adapted to Israel^49^. It comprises ten items rated on a Likert scale ranging from 1 (definitely not true) to 5 (definitely true). A sample item is: “I worry that others have more positive experiences than me.” Scores are computed as the mean of the items, resulting in a range of 1 to 5. In the present study, Cronbach’s alpha for the total score was 0.83.

The *ENRICH Romantic Relationship Satisfaction Inventory*^82^ was translated into Hebrew and adapted to the Israeli context^83^. The questionnaire is used to measure romantic relationship satisfaction. The Hebrew instrument, which consists of ten items, evaluates challenges and strengths within the relationship across nine dimensions (personality issues, communication, conflict resolution, financial management, leisure activities, sexual relationships, children and parenting, extended family and friends, and gender role attitudes). A sample item is: “I’m very pleased with my partner’s personality and personal habits.” Participants respond on a Likert scale spanning from 1 (not at all true for me) to 7 (very true for me). A higher score reflects greater satisfaction. Scores are computed as the mean of the items. Research has demonstrated test–retest reliability and internal consistency of the subscales and overall assessment^82^. In the current study, Cronbach’s alpha for the entire sample was 0.82.

*The Loneliness in Intimate Relationships (LIRS)* scale^68^ was initially developed in Hebrew and comprises 14 items. Respondents are prompted to evaluate the degree to which they experience feelings of loneliness within their romantic relationship (sample item: “I feel hurt.”) The instrument is divided into three subscales: detachment (6 items), harm (4 items), and guilt (4 items). Subscale scores are generated by calculating the mean of the corresponding item ratings, where higher scores indicate heightened sensations of detachment, hurt, and guilt. Given substantial intercorrelations among the subscales, a total mean score was computed in the present study. Respondents’ answers are appraised along a Likert scale, spanning from 1 (does not describe my experience at all) to 6 (totally describes my experience). Internal consistency, assessed by Cronbach’s alpha, was notably robust (0.93).

*Demographic questionnaire* A questionnaire was crafted for the current study, encompassing pertinent background details such as gender, age, relationship status, and level of education.

### Procedure

After obtaining ethics approval from the ethics committee of the college (IRB) (YVC EMEK 63-2023), a survey company was employed to distribute the questionnaires to an online systematic sample. The survey was performed in accordance with the guidelines and regulations. Informed consent was obtained from all participants by signing the initial page of the first questionnaire. Questionnaire completion was voluntary, with participants assured of anonymity and confidentiality. They were also informed of their right to cease participation at any point.

### Statistical analyses

Data were analyzed using SPSS version 29. The examination of internal consistencies led to the construction of variables through item means. Descriptive statistics, including means and standard deviations, were employed to portray the study variables. Pearson correlations were computed to assess relationships among variables. Similarly, Pearson correlations and independent t-tests were executed to explore connections between the study variables and demographic characteristics.

To evaluate the study model, path analysis was employed, utilizing AMOS version 29. Model fit was evaluated using metrics including Chi-square, cmin/df, NFI, NNFI, CFI, and RMSEA. The independent variables were gender and the DoS dimensions. Age and level of education were controlled for. Correlations among demographic variables, DoS dimensions, and mediators were examined within each group of variables. The mediation analysis was integrated into the path analysis, employing 5,000 bootstrap samples with bias-corrected 95% confidence intervals. Variables were standardized. Finally, the interactions between gender and the study variables were examined, with multiple regression analyses, to assess whether the model associations differed by gender.

### Ethical approval

Ethics committee approval was obtained for the study. All procedures in this study involving human participants were conducted in accordance with the ethical standards of the 1975 Helsinki Declaration and were approved by the research team’s college ethics committee.

### Informed consent

Informed consent for participation was obtained before completion of the survey. Participants were sent a form containing information regarding the purpose of the study and voluntary participation.

## Results

### Descriptive results

Table 2 shows mean scores and correlations for the study variables. Average scores hovered near midpoint for all variables, although they were somewhat higher for romantic relationship satisfaction and I-position and relatively low for emotional cutoff. Most correlations were significant. Phubbing was positively associated with FoMO, emotional reactivity and fusion with others, and negatively associated with I-position. FoMO was positively related to loneliness in intimate relationships, emotional reactivity, emotional cutoff, and fusion with others, and negatively related to I-position. Romantic relationship satisfaction was negatively associated with loneliness and emotional cutoff, and positively associated with I-position. Loneliness in intimate relationship was positively associated with all three negative dimensions of DoS.

**Table 2:** Means, standard deviations, and correlations for the study variables (N = 431).

	*M* (*SD*)	1	2	3	4	5	6	7	8
1. Phubbing	2.76 (0.63)	1							
2. FoMO	2.51 (0.72)	.31***	1						
3. Romantic relationship satisfaction	4.02 (0.57)	.01	− .07	1					
4. Loneliness	2.66 (1.13)	.12	.28***	− .40***	1				
5. I-position	3.92 (0.85)	− .18***	− .25***	.20***	− .12	1			
6. Emotional reactivity	3.52 (1.06)	.23***	.46***	− .02	.37***	− .43***	1		
7. Emotional cutoff	2.42 (0.82)	.13	.30***	− .38***	.51***	− .07	.33***	1	
8. Fusion with others	3.62 (0.82)	.21***	.43***	.07	.22***	− .35***	.68***	.18***	1

Range = 1–5 for phubbing and FoMO; 1–7 for romantic relationship satisfaction; 1–6 for loneliness and DoS dimensions.

***p < .001. Bonferroni correction: *p* ≤ .001.

Several gender differences emerged, as shown in Table 3. Females exhibited higher levels of phubbing than males, as well as higher levels of emotional reactivity and fusion with others, while males exhibited higher levels of I-position than females.

**Table 3:** Means, standard deviations and t-tests for the study variables (N = 431).

	Female *M* (*SD*)	Male *M* (*SD*)	*t*(429)	*p*	*d*
Phubbing	2.82 (0.64)	2.66 (0.61)	2.47	**.014**	0.25
FoMO	2.56 (0.74)	2.42 (0.67)	1.93	.055	0.19
Romantic relationship satisfaction	4.05 (0.52)	3.96 (0.65)	1.52	.130	0.16
Loneliness	2.61 (1.08)	2.75 (1.2)	− 1.24	.216	− 0.12
I-position	3.77 (0.82)	4.21 (0.84)	− 5.31	** < .001**	− 0.54
Emotional reactivity	3.77 (1.02)	3.04 (0.96)	7.23	** < .001**	0.73
Emotional cutoff	2.38 (0.81)	2.49 (0.83)	− 1.33	.185	− 0.13
Fusion with others	3.76 (0.8)	3.35 (0.79)	5.15	** < .001**	0.52

In addition, two demographic factors were found to be associated with the study variables (see supplementary material). Age was negatively associated with FoMO, romantic relationship satisfaction, loneliness in intimate relationships, and fusion with others. Participants with a college education exhibited higher levels of phubbing and lower levels of loneliness in intimate relationships and emotional cutoff than those with only a high school diploma. Consequently, these two demographic factors were employed as control variables in subsequent analyses.

### Analyses of the study model

**Figure 2:**
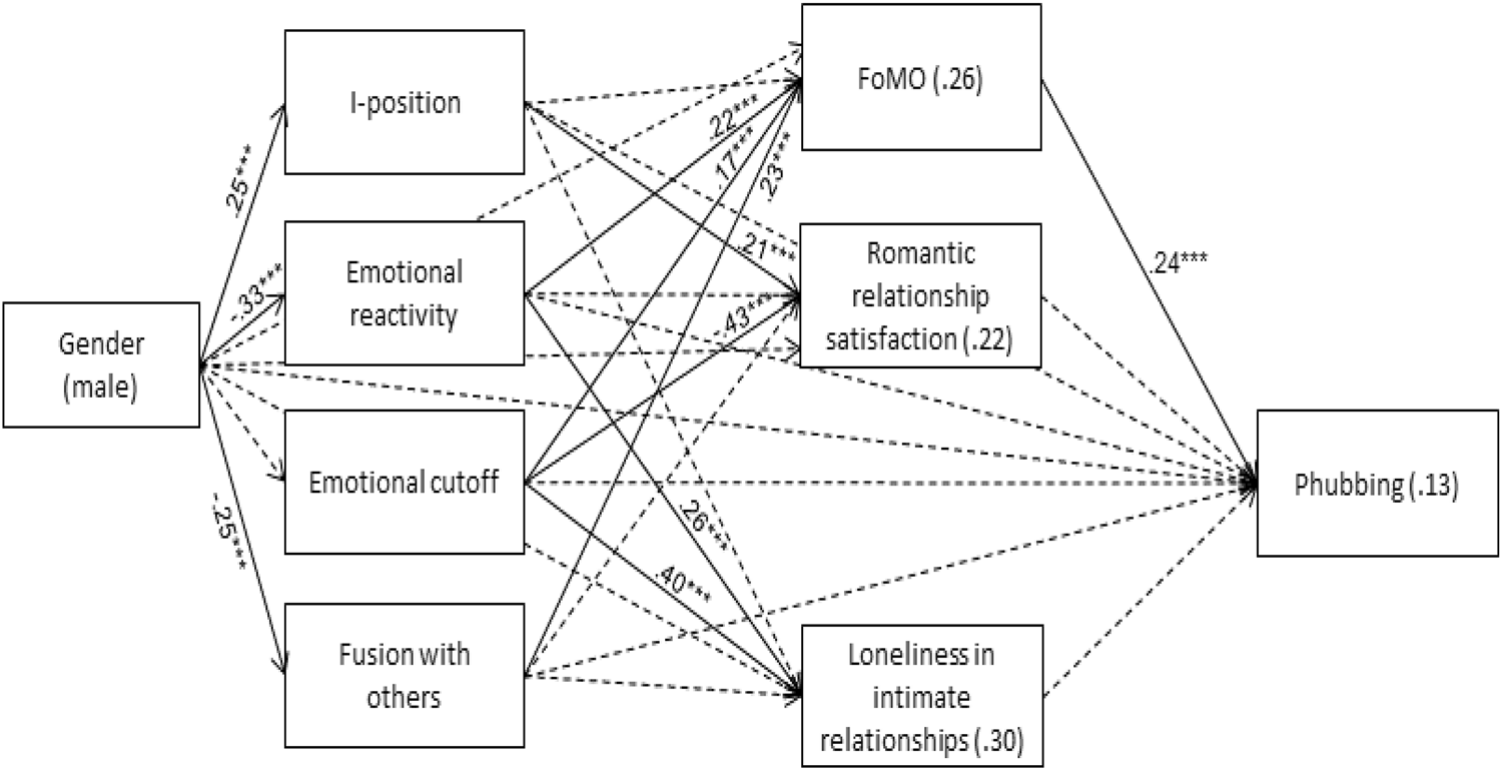
Path Analysis for FoMO, romantic relationship satisfaction, and loneliness in intimate relationships as mediating between differentiation of self and phubbing. *Note R*^2^ values appear within rectangles; significant β values (standardized regression coefficients) appear above arrows. Solid lines indicate significant associations; dotted lines display non-significant associations. *p < .05; **p < .01; ***p < .001.

Path analysis of the study model used gender (1—males, 0—females) and the four DoS dimensions as independent variables. FoMO, romantic relationship satisfaction, and loneliness in intimate relationships were the mediators, and phubbing was the dependent variable. Age and level of education (1—college, 0—high school) were controlled for. Mediation was examined within the path analysis, with a bootstrapping of 5,000 samples and a bias-corrected 95% confidence interval. For enhanced clarity, significant path values are presented in Fig. 2. The model was found to fit the data: χ^2^(9) = 9.97, *p* = .353, cmin/df = 1.11, NFI = .991, NNFI = .995, CFI = .999, RMSEA = .016.

The path analysis results (Fig. 2) revealed several significant associations. First, males scored higher than females on I-position, while females scored higher than males on emotional reactivity and fusion with others. Higher emotional reactivity, emotional cutoff, and fusion with others were associated with higher FoMO. Higher I-position and lower emotional cutoff were associated with higher romantic relationship satisfaction. Higher emotional reactivity and emotional cutoff were associated with a higher level of loneliness in intimate relationships. Finally, increased FoMO was associated with higher phubbing. However, romantic relationship satisfaction and loneliness in intimate relationships had non-significant associations with phubbing (β = .09, *p* = .107 and β = .04, *p* = .487, respectively; not shown in figure).

Results of the path analysis thus suggest that FoMO acts as a mediator in associations between the dimensions of DoS and phubbing. However, associations between romantic relationship satisfaction and phubbing, as well as loneliness in intimate relationships and phubbing, were non-significant, indicating no apparent indirect effects involving them.

The indirect effects observed for FoMO are detailed in Table 4. Notably, higher emotional reactivity, emotional cutoff, and fusion with others were associated with heightened FoMO, which in turn was connected to increased phubbing behavior. This indirect effect was non-significant for I-position, as there was no discernible link between I-position and FoMO. Finally, all interactions between gender and the study variables in predicting phubbing were non-significant (*p* = .069 to *p* = .860), as were all interactions between gender and the study variables in predicting the mediating variables (*p* = .132 to *p* = .909). Thus, the results for the study model may be regarded as quite similar for males and females.

**Table 4:** Indirect effects for phubbing, with differentiation of self and FoMO (N = 431).

Predictor	Mediator	Dependent variable	Effect (*SE*)	*p*	95% CI
I-position	FoMO	Phubbing	− 0.01 (0.01)	.071	− 0.03, 0.01
Emotional reactivity	FoMO	Phubbing	0.04 (0.01)	** < .001**	0.02, 0.07
Emotional cutoff	FoMO	Phubbing	0.03 (0.01)	** < .001**	0.01, 0.05
Fusion with others	FoMO	Phubbing	0.03 (0.01)	** < .001**	0.01, 0.06

## Discussion

In this study, we have delved into the mediating roles of FoMO, romantic relationship satisfaction, and loneliness in intimate relationships in the connection between DoS and phubbing behavior. On the whole, findings indicate that FoMO indeed served as a mediator in the link between three dimensions of DoS (emotional reactivity, emotional cutoff, and fusion with others) and phubbing behavior. However, the hypothesized mediating role of romantic relationship satisfaction and loneliness in intimate relationships did not reach statistical significance. Nonetheless, these latter two variables did yield associations with DoS.

The findings provide partial support for Hypothesis 1, suggesting that FoMO mediates the relationship between DoS and phubbing. This aligns with research suggesting that FoMO mediates the association between personality characteristics and phubbing (e.g.^30^). The present results provide novel insights into how the association between DoS and FoMO impacts the misuse of social media (i.e., phubbing), suggesting that uncertainties about interpersonal relationships can intensify apprehension about missing out on opportunities, motivating a heightened reliance on social media to alleviate such unease. The heightened FoMO might, in turn, contribute to increased utilization of online platforms, leading to a rise in phubbing behavior.

The association between phubbing and personality traits can also be elucidated through the framework of the Uses and Gratifications Theory. This theory suggests that people use social media to fulfill various needs, including maintaining relationships, seeking entertainment, and gaining admiration^34–36^. Considering the inherent uncertainty within interpersonal relationships and in self-perception among individuals with lower levels of DoS, such people might find themselves spending prolonged periods navigating social networks and intensifying their Internet and social media usage, experiencing high FoMO levels. Our findings align with the assumption that individuals characterized by poor DoS often exhibit traits like self-doubt, anxiety, and uncertainty in interpersonal relationships. These traits are likely to contribute to an increased sense of FoMO. Such individuals may thus use social media platforms to decrease their anxiety and fulfil their desires^84^. Furthermore, as individuals with high FoMO are prone to anxiety about missing out on others’ activities^85^ and tend to engage in frequent self-comparisons^86^, they may extensively incorporate social media into their daily lives^87^, potentially increasing instances of phubbing^34^.

Contrary to research that established a link between romantic relationship satisfaction and phubbing (e.g.^1,29,88,89^), the current study did not find such satisfaction to be a mediating factor or directly associated with phubbing, similar to recent findings that 429 adults in China reported a lack of significant connection between relationship satisfaction and partner phubbing^90^. Wang and colleagues proposed that this link might be influenced by self-esteem, suggesting a potential moderating role of self-esteem within this particular context.

While the reasons underlying the lack of significant association between romantic relationship satisfaction and phubbing in this study require further investigation, several explanations can be tentatively offered. The first concerns diverse coping strategies. People employ a range of coping mechanisms when facing relationship difficulties; while some might resort to phubbing, others might seek alternative solutions, such as engaging in activities with family and friends or preferring solitude. This diversity in coping styles could lead to varied expressions of romantic relationship satisfaction and phubbing behavior among couples, making the relationship between these factors less clear cut.

A second possibility is related to the duration of the relationship. As most participants in the current study were young adults, it is conceivable that their relationships were still in the early stages and relatively free from strain. This could impact phubbing behaviors.

Finally, another possible explanation concerns a unique shift in the study focus. Unlike existing research, this study had participants report their own phubbing behavior, rather than focusing on instances of being phubbed. Moreover, whereas prior studies primarily investigated the effect of phubbing on romantic relationship satisfaction, the current one explored the lack of romantic relationship satisfaction as a possible contributor to phubbing. All of this could have implications for the observed outcomes.

While romantic relationship satisfaction and loneliness in intimate relationships were not directly related to phubbing or play a mediating role, our findings do underscore their connection to DoS, supporting previous studies^22,91^. These findings corroborate Bowen’s^14,92^ theoretical arguments that well-differentiated individuals can maintain stable and fulfilling intimate connections without compromising their basic selves, while poorly differentiated individuals may grapple with feelings of isolation due to difficulties in effectively balancing intimacy and autonomy^20^. The two dimensions of DoS that predicted romantic relationship satisfaction, i.e., I-position and emotional cutoff, reflect (from two different sides of the coin) the capacity for open communication, expressing emotions, desires, and needs directly. It is possible that dissatisfied partners have difficulty engaging in sincere communication, which inhibits both the expression of their own feelings and their ability to listen to their partner’s feelings.

Our second hypothesis suggested that women would report higher levels of emotional reactivity, fusion with others, and FoMO, while men would report higher levels of emotional cutoff and phubbing. This hypothesis was partially supported: females did report higher levels of emotional reactivity, fusion with others, and phubbing, while males reported higher levels of I-position, partially supporting previous studies (e.g.^12,93^). It should be noted that no gender difference was found for FoMO. It has been suggested^20^ that, due to distinct socialization processes, there are differences in how women and men express their emotions. Women may feel more comfortable discussing and displaying feelings of anxiety and fear, as societal norms generally permit and even encourage this kind of emotional expression. In our study, men reported higher levels of I-position, indicating that they perceive themselves as more capable of standing up for their own desires than women do. Societal norms are thought to promote assertiveness in men while discouraging it in women, resulting in observed differences. From a young age, children are frequently exposed to gender-based societal norms that prescribe specific behaviors. These expectations can shape the development of assertiveness.

In addition to the above, men reported greater loneliness within intimate relationships than women. This may suggest that men’s reluctance to seek help might contribute to higher levels of loneliness^94^.

Contrary to earlier research (e.g.^95,96^) and to the second hypothesis, women reported higher levels of phubbing than men. It is possible that women are more likely to recognize and report specific behaviors. This finding may also reflect a change in cultural expectations about phone use in social settings, impacting reporting tendencies, especially among young women. A comprehensive investigation that considers these factors, as well as qualitative insights and cultural context, is essential for a more profound understanding of this issue.

### Limitations

This study has several limitations. Firstly, the sample primarily consists of young adults, which may hinder the extent to which the findings can be generalized to other age groups. While young adults have a greater propensity for smartphone use, expanding the age range of the sample would be prudent to ensure a more diverse representation. Secondly, given that the study has a cross-sectional design, the obtained results do not establish a definitive causal connection among the examined variables. To enhance comprehension and rigor, future investigations employing alternative methodologies, such as semi-structured interviews, or longitudinal designs, may provide more comprehensive insights. Furthermore, it is possible that the use of different data collection methods would have avoided the common method bias. Thirdly, the inclusion of additional variables, such as ethnicity and socioeconomic status, which might influence or moderate the relationships among the study variables, could yield valuable insights into the phenomenon of phubbing. Finally, it should be noted that, as participants filled out questionnaires for all independent, mediating, and dependent variables, the possibility of a common method bias was created.

### Contributions

Notwithstanding its limitations, the study makes important contributions to the literature. Given the massive global increase of smartphone usage^29^, the significance of phubbing will continue to grow, increasing the need for comprehensive research to better understand the impact of this behavior and its underlying factors. The current study contributes to a deeper comprehension of the psychological factors underlying phubbing behaviors, which remains a relatively novel concept.

The established link between DoS and phubbing is also noteworthy. Poorly differentiated individuals seem to be at higher risk of engaging in phubbing behaviors through the mediation of FoMO. This outcome is pivotal for identifying focus groups for future research aimed at reducing phubbing behaviors. Furthermore, the finding that FoMO predicts phubbing has crucial implications for understanding the diverse motivations driving phubbing. We propose that FoMO might act as a catalyst, prompting individuals to experience anxiety, which in turn leads them to resort to phubbing as a coping mechanism.

In addition, the current study contributes to the literature because of its unique focus—namely, examining participants’ phubbing behavior, rather than whether they themselves were being phubbed. Thus, our findings illuminate the characteristics of individuals who engage in phubbing.

The research also has some practical implications. By comprehending factors that may escalate phubbing behavior, psychologists, psychiatrics, educational counselors, and family therapists can offer assistance to couples with high levels of phubbing. For instance, interventions aimed at enhancing DoS, which may reduce FoMO, could potentially lead to a reduction in phubbing. This knowledge may generate practical strategies for improving the quality of relationships in the context of digital device usage. At the same time, it is crucial to customize counseling specifically for women and men, considering the gender differences identified in the current study.

### Supplementary Information

Supplementary Information.

## Data Availability

The datasets generated during and analyzed during the current study are available from the corresponding author upon reasonable request.
