# Supplementary material for: Exploring the personality and relationship factors that mediate the connection between differentiation of self and phubbing
Source: Sci Rep. 2024 Mar 19;14:6572. doi: 10.1038/s41598-024-55560-1 (PMC10951228; doi:10.1038/s41598-024-55560-1)
Supplement: Supplementary file 1 — Supplementary Information. [file 41598_2024_55560_MOESM1_ESM.docx]

**Supplementary material**

## Correlations Between Study Variables and Demographic Factors

Participants’ age was negatively associated with FoMO (r = -.18, p < .001), romantic relationship satisfaction (r = -.22, p < .001), loneliness in intimate relationships (r = -.13, p = .007), and fusion with others (r = -.20, p < .001). In other words, younger participants reported a greater degree of FoMO, romantic relationship satisfaction, loneliness in intimate relationship, and fusion with others. Age was also strongly associated with relationship duration (r = .76, p < .001), relationship status (r = .52, p < .001) and parenthood (r = .74, p < .001).

Participants with a college education reported higher levels of phubbing than high school graduates [M = 2.82, SD = 0.63 vs M = 2.68, SD = 0.63; t(429) = 2.20, p = .029]. They also reported lower levels of loneliness in intimate relationships and emotional cutoff than those with a high school diploma [loneliness: M = 2.51, SD = 1.05 vs. M = 2.87, SD = 1.20; t(338.77) = -3.18, p = .002; emotional cutoff: M = 2.32, SD = 0.74 vs. M = 2.57, SD = 0.90; t(322.83) = -3.10, p = .002].
